# Salinity Sensor Using a Tapered Polarization-Maintaining Fiber-Based Sagnac Loop in a Fiber Ring Laser with Support Vector Regression for Improved Accuracy

**DOI:** 10.3390/s26123953

**Published:** 2026-06-22

**Authors:** Weihao Lin, Zihan Huang, Keyu Cai, Mingkun Zhang, Renan Xu, Yuhui Liu

**Affiliations:** 1 The Higher Educational Key Laboratory for Flexible Manufacturing Equipment Integration of Fujian Province, Xiamen Institute of Technology, Xiamen 361021, China; hzhan_reiko@163.com (Z.H.); 13788869098@163.com (K.C.); zhangmingkun@xit.edu.cn (M.Z.); xurenan@xit.edu.cn (R.X.); 2 Institute for Photonics Technology, Quanzhou Normal University, Quanzhou 362000, China; 3 Fujian Provincial Key Laboratory for Advanced Micro-Nano Photonics Technology and Devices, Quanzhou 362000, China; 4 Fujian Provincial Collaborative Innovation Center for Ultra-Precision Optical Engineering and Applications, Quanzhou 362000, China; 5 Fujian Provincial University Engineering Research Center for Intelligent Optical Fiber Sensing and Testing Technology, Quanzhou 362000, China

**Keywords:** fiber ring laser, salinity sensing, Sagnac loop, support vector regression

## Abstract

This paper proposes and experimentally demonstrates a fiber ring laser (FRL) salinity sensing system based on a Sagnac loop (SL) formed by a tapered polarization-maintaining fiber (TPMF). The operating principle is that salinity modulates the birefringence of the polarization-maintaining fiber (PMF), causing a shift in the interference wavelength of the SL transmission spectrum, while the FRL narrows the optical spectrum and enhances the signal-to-noise ratio (SNR). In the experiment, the SL consists of a 20-cm-long PMF with a tapered waist diameter of 10.86 μm. Over the salinity range of 0‰ to 30‰, the sensitivity of the laser-based sensing system is 97 pm/‰, which agrees well with the 93 pm/‰ sensitivity obtained using a broadband light source (BBS), and the salinity exhibits a good linear relationship with the wavelength shift, with a coefficient of determination (R^2^) of 0.997. Meanwhile, the ring laser cavity improves the SNR of the sensing system from 22 dB to approximately 54 dB, and compresses the 3-dB bandwidth from 1.75 nm to 0.06 nm. Further adopting the support vector regression (SVR) algorithm for linear regression modeling of the spectral data, the results show that the mean absolute error (MAE) decreases from 0.50‰ to 0.04‰, the root mean square error (RMSE) decreases from 0.54‰ to 0.11‰, and R^2^ reaches as high as 0.99988. To the best of our knowledge, this is the first work that combines salinity laser sensing with an artificial intelligence algorithm. The proposed sensor leverages the narrow linewidth and high SNR advantages of the FRL together with the high-precision linear fitting capability of the SVR algorithm, achieving significantly improved accuracy for salinity measurement compared to conventional spectral demodulation.

## 1. Introduction

Salinity is a key parameter in seawater, estuaries, lakes, and aquaculture environments, playing a vital role in marine ecological monitoring, climate research, seawater desalination, and the food industry [1,2,3]. Accurate and reliable salinity measurement is therefore essential for these applications. Fiber optic sensors have been widely investigated for salinity monitoring due to their compact size, corrosion resistance, and immunity to electromagnetic interference; however, their practical application remains limited compared to traditional electrical conductivity sensors [4,5]. However, the majority of fiber-optic salinity sensors rely on broadband sources (BBSs) combined with interferometric structures [6]. Owing to the broad bandwidth and low signal-to-noise ratio (SNR) inherent to BBSs, such systems can easily lead to large measurement errors.

In recent years, fiber ring lasers (FRLs) have emerged as a compelling alternative to BBSs in fiber-optic sensing systems [7]. By incorporating a gain medium and a wavelength-selective element within the laser cavity, an FRL can generate narrow-linewidth and high-SNR output, enabling much more precise detection of spectral shifts. This intrinsic advantage has led to successful applications of FRL-based sensors for temperature, strain, and refractive index measurements with improved resolution and repeatability [8,9,10].

For salinity sensing, a few exploratory studies have adopted FRL configurations [11]. In 2014, Meng et al. reported a laser salinity monitoring system based on multimode interference using a no-core fiber (NCF), but the weak evanescent field resulted in low sensitivity [12]. In 2019, Xu et al. proposed an intensity-modulated fiber laser salinity sensor by cascading a fiber Bragg grating (FBG) and a Fabry–Perot cavity, achieving a sensitivity of 0.1 W/‰; however, the cascaded structure was highly sensitive to mechanical vibrations, compromising repeatability [13]. In 2022, Zhao et al. embedded an SL formed by a tapered side-hole fiber (SHF) into an FRL cavity [14]. Although this scheme realized in-cavity salinity detection, its sensitivity varied significantly across different salinity ranges, leading to poor linearity. In summary, existing laser-based salinity sensors still suffer from issues such as low sensitivity, mechanical instability, or nonlinear response. Therefore, there remains an urgent need to develop an FRL-based salinity sensor that simultaneously offers a high SNR, good linearity, and high measurement accuracy.

However, simply optimizing the optical structure cannot satisfy all these performance requirements at once. In recent years, combining machine learning algorithms with fiber-optic sensing technology has become an effective approach to improve measurement accuracy [15,16,17]. Specifically, for salinity sensing, several studies have attempted to introduce machine learning [18,19]. Xiao et al. used a Bayesian regularized back-propagation network for demodulating the data, achieving a mean relative error (MRE) of 0.766% [20]. Majee et al. used an ANN to reduce the RMSE of salinity by a factor of 500 [21]. However, the above works rely on wavelength modulation schemes with BBSs, where the limited spectral quality restricts the improvement in accuracy that algorithms can provide. To the best of our knowledge, no work has yet reported the combination of a fiber-optic laser-based salinity sensor with a machine learning algorithm.

In this work, we propose and experimentally demonstrate an FRL-based salinity sensing system using an SL formed by a TPMF. The sensor embeds the TPMF-based SL into an FRL cavity, leveraging the narrow linewidth and high SNR of the laser cavity to improve spectral quality. It further employs an SVR algorithm for linear regression modeling of the spectral data to enhance the accuracy of salinity prediction. In the experiment, the SL is fabricated from a 20-cm-long PMF with a tapered waist diameter of 10.86 μm. Over the salinity range of 0‰ to 30‰, the sensitivity of the laser sensing system is 97 pm/‰, which agrees well with the 93 pm/‰ sensitivity obtained using a BBS. Meanwhile, the ring laser cavity increases the SNR from 22 dB to approximately 54 dB and compresses the 3-dB bandwidth from 1.75 nm to 0.06 nm. After applying the SVR algorithm, the MAE decreases from 0.50‰ to 0.04‰, the RMSE decreases from 0.542‰ to 0.11‰, and R^2^ reaches 0.99988. To the best of our knowledge, this is the first work that combines salinity laser sensing with an artificial intelligence algorithm. The proposed system is expected to play an important role in aquaculture water quality management and estuarine ecological monitoring.

## 2. Working Principle and Experimental Setups

**Figure 1:**
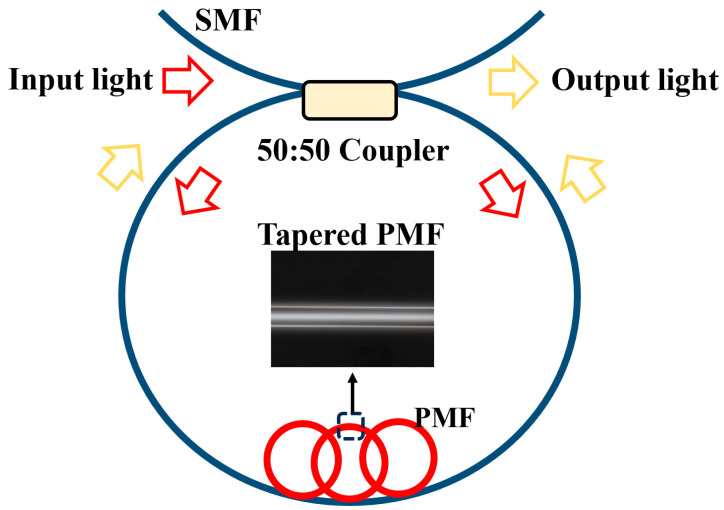
Schematic diagram of the operating principle of the SL for salinity monitoring (Red arrow: Input light; Yellow arrow: Output light).

Figure 1 illustrates the basic operating principle of the SL. The loop is formed by connecting a section of PMF (PM1017-B, Yangtze, Wuhan, China) to the output ports of a 3 dB coupler. When light is launched into the coupler, it is split into two beams of equal intensity that travel in opposite directions. After propagating along the PMF for one round trip, the two beams return to the coupler and interfere with each other. The PMF possesses two orthogonal polarization axes, and the difference in the refractive index between them is defined as the birefringence B. Letting the PMF length be L, the phase difference between the two counter-propagating waves is given by [22]:

(1) $$∆\varphi =\frac{2\pi BL}{\lambda }$$

The transmittance T of the SL is given by:

(2) $$T\left(\lambda \right)=\frac{1}{2}\left[1-\cos\left(\frac{2\pi BL}{\lambda }\right)\right]$$

The wavelength λ corresponding to the transmission minimum satisfies [22]:

(3) $$\lambda_{m}=\frac{2BL}{2m+1},\;m=0,1,2,\cdots$$

**Figure 2:**
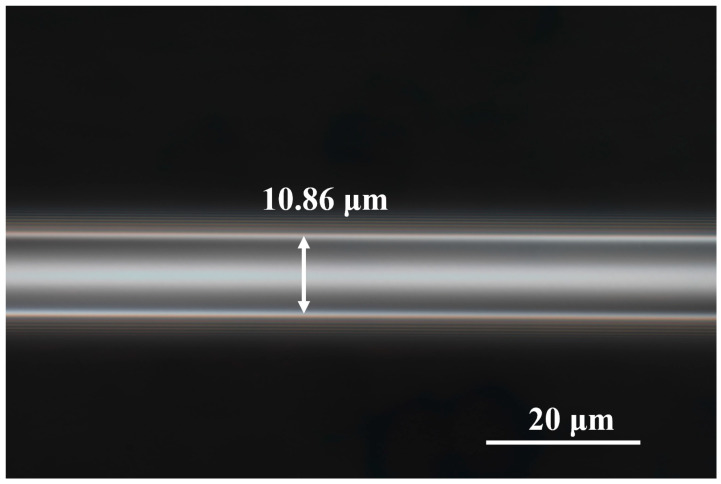
Microscopic image of the TPMF.

After the PMF is tapered (AFBT-8000, Shandong Kaipule Optoelectronic Technology Co., Ltd., Tai’an, China), the waist diameter reaches 10.86 μm in our experiment, as shown in Figure 2 (LV100ND, Nikon Corporation, Tokyo, Japan). At this stage, the tapered region becomes a thin dielectric waveguide. The optical field propagating inside the fiber is no longer tightly confined. Instead, it extends outside the fiber in the form of an evanescent wave, directly interacting with the surrounding salt solution. A change in salinity alters the refractive index of the salt solution, which generally increases monotonically with rising salinity. This refractive index variation is fed back to the propagating optical fields of the fiber through the evanescent wave coupling mechanism, leading to a corresponding change in the effective refractive index of the fiber. Since the birefringence B of a PMF is essentially the difference in effective refractive indices between its two orthogonal polarization axes, the salinity-induced effective index variation ultimately results in a modulation of the birefringence B. The total length of the PMF used in the experiment is 20 cm, which provides sufficient birefringence accumulation length for the SL to produce a resolvable wavelength shift. Over a small range of salinity variation, the birefringence B is approximately linear with the salinity S, which can be expressed as:

(4) $$B\left(S\right)=B_{0}+k_{B}\cdot S$$

where $B_{0}$ is the birefringence at a salinity of 0‰, and $k_{B}$ is the salinity sensitivity coefficient. The magnitude of this coefficient mainly depends on the evanescent field intensity of the tapered fiber and the response efficiency of the fiber material to changes in the refractive index of the surrounding medium.

**Figure 3:**
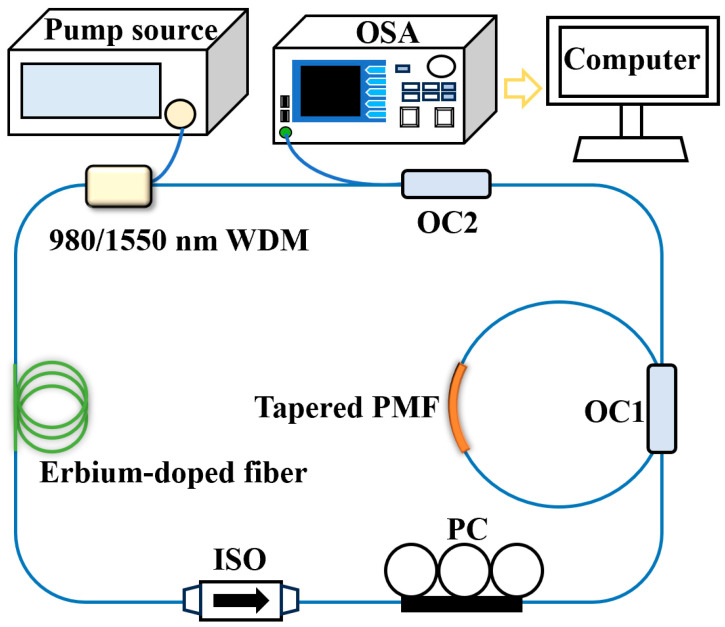
Experimental setup of the fiber ring laser salinity sensor (OC: optical coupler).

Figure 3 illustrates the experimental setup of the proposed salinity sensor based on an FRL. The entire experiment was conducted in a laboratory environment with a constant temperature of 25 °C to eliminate the influence of ambient temperature fluctuations on the fiber birefringence and laser stability. A pump laser source (MP14, Micro photons Technology Co., Ltd., Shanghai, China) launched its output into the ring laser cavity through a wavelength-division multiplexer (WDM), which combines the pump and signal light while isolating residual pump light. The pump light first entered a segment of erbium-doped fiber (EDF, Thorlabs ER30-4/125, Thorlabs, Newton, NJ, USA) with a length of 80 cm, where the gain medium absorbed pump energy, excited erbium ions to higher energy levels, and generated amplified spontaneous emission in the 1550 nm band. Subsequently, the optical signal passed through an optical isolator (ISO) and a polarization controller (PC). The ISO enforced unidirectional light propagation inside the cavity, preventing back reflections that could cause mode competition or instability. The PC adjusted the polarization state of the circulating light to optimize the interference contrast of the SL. After that, the signal arrived at a 50:50 coupler, which split it equally into two paths directed into the SL. The SL consisted of a 20-cm-long TPMF with a waist diameter of 10.86 μm. Acting as a wavelength-selective element, the SL exhibited a transmission spectrum that shifted with salinity variations. Light traveling both clockwise and counter-clockwise over one round trip inside the SL interfered at the 50:50 coupler and then re-entered the main ring cavity. A 90:10 output coupler was also placed in the cavity: 90% of the optical energy remained inside the cavity to sustain laser oscillation, while the remaining 10% was coupled out to an optical spectrum analyzer (OSA, AQ6370D, YOKOGAWA, Tokyo, Japan). The OSA recorded the transmission spectrum and transferred the data to a computer. The computer first pre-processed the spectra and then performed linear regression modeling on the relationship between the spectral data and salinity using an SVR algorithm, thereby achieving high-precision retrieval of salinity values.

In the FRL constructed in this work, the SL simultaneously serves as both the sensing element and the intracavity wavelength-selective filter. The laser output wavelength is determined as follows: The transmission spectrum of the SL exhibits periodic interference peaks. When the net gain at a specific wavelength in the cavity first reaches the laser oscillation threshold, that wavelength becomes locked to the corresponding interference peak and starts to oscillate. When the external salt solution contacts the tapered region of the fiber, the evanescent field effect modulates the effective refractive index of the PMF, causing an overall shift of the SL transmission spectrum [23]. Since the laser oscillation wavelength is always locked to the peak position of the transmission spectrum, the laser wavelength follows the shift of the interference peak synchronously.

In the experiment, it was observed that at low salinity concentrations, the laser oscillates simultaneously at two interference peaks. As the salinity increases, the dual-peak oscillation gradually converts into single-peak oscillation. This phenomenon originates from mode competition induced by the homogeneous broadening characteristic of the EDF [24]. The homogeneous broadening of the EDF dominates, causing gain competition among different longitudinal modes and making it difficult to achieve stable multi-wavelength output. Initially, the gain conditions of two adjacent peaks in the SL transmission spectrum are comparable, and both satisfy the oscillation threshold, so the two peaks oscillate simultaneously. When the salinity increases, the entire transmission spectrum shifts, and the transmission intensity of one interference peak decreases relatively, so its net gain gradually falls below the oscillation threshold. As the salinity continues to increase, one wavelength mode gains an absolute advantage in the competition, and the other mode is completely suppressed. Consequently, only one oscillation peak is eventually observed.

It is worth noting that although the number of oscillation peaks changes, the wavelength shift of the two peaks remains nearly the same under different salinity concentrations. This is because adjacent interference peaks in the SL transmission spectrum have almost the same free spectral range (FSR), and the salinity-induced refractive index change produces almost equal wavelength shifts for each interference peak. Therefore, the salinity-induced shift of the interference peak directly determines the movement of the laser wavelength, while the change in the number of oscillation peaks is naturally determined by the gain competition mechanism. The above mechanism ensures that the proposed sensor always maintains a stable wavelength–salinity linear response.

**Figure 4:**
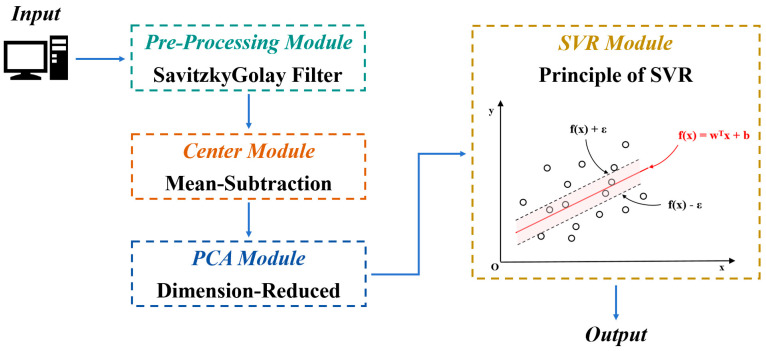
Principle and flowchart of salinity prediction accuracy enhancement based on the SVR algorithm.

Figure 4 illustrates the principle and flowchart of accuracy enhancement based on the SVR algorithm. The total dataset comprises seven salinity groups with concentrations ranging from 0‰ to 30‰ at intervals of 5‰. For each salinity level, 125 spectra are recorded, resulting in a total of 875 samples. Each CSV file is read and converted into a one-dimensional spectral sequence, which serves as the input feature vector for one sample. According to the data parsing logic in the script, the feature length of a single sample is 5501. To evaluate the generalization performance of the model, the dataset is divided into a training set, a validation set, and a test set. Specifically, the training set contains 525 samples, the validation set contains 175 samples, and the test set contains 175 samples. The training set is used for model fitting, the validation set for model selection and parameter tuning, and the test set for final evaluation of the model’s predictive ability on unseen samples.

To reduce random noise in the raw spectral signals while preserving peak shapes and local trends, each sample is first processed by a Savitzky–Golay smoothing filter. According to the settings in this work, the smoothing window length is 7, the polynomial order is 3, and the derivative order is 0, meaning only smoothing is performed without differentiation. This method applies local polynomial fitting to smooth the data, effectively suppressing high-frequency noise while maintaining the structural features of the original signal. After smoothing, each sample is centered. The purpose of centering is to remove the overall offset of each sample, making the data distribute around a zero mean, thereby reducing the influence of baseline drift and overall intensity variations on subsequent modeling. After this step, the focus of sample modeling shifts from absolute amplitude values to curve shapes and variation patterns. Because the original input features are high-dimensional and strong correlations usually exist among different wavelength points, principal component analysis (PCA) is further applied to the centered data for dimensionality reduction [25]. The core idea of PCA is to find a set of new orthogonal axes through linear transformation such that the projected features retain as much of the original variance information as possible. Through this step, the original high-dimensional spectrum is compressed into a few principal components, providing a more compact and effective input for SVR modeling.

After PCA dimensionality reduction, the principal components are fed into an SVR model for final prediction. The goal of SVR is to learn the nonlinear mapping relationship between the input features and the salinity value while controlling the model complexity. The working principle of SVR is also illustrated in Figure 4, adopting the ε-insensitive loss idea; i.e., no penalty is given when the prediction error falls within the ε-tube, and constraints are imposed via slack variables only when the error exceeds this tube. The prediction function of SVR can be written as [25]:

(5) $${min}_{\omega ,b,\xi_{i},\xi_{i}^{*}}\frac{1}{2}{\left\|w\right\|}^{2}+C\sum_{i=1}^{n}\left(\xi_{i}+\xi_{i}^{*}\right)$$

The constraints can be expressed as:

(6) $$y_{i}-\left(w^{T}\phi \left(z_{i}\right)+b\right)\leq \varepsilon +\xi_{i}$$

(7) $$\left(w^{T}\phi \left(z_{i}\right)+b\right)-y_{i}\leq \varepsilon +\xi_{i}^{*}$$

(8) $$\xi_{i},\;\xi_{i}^{*}\geq 0$$

where w and b denote the normal vector and bias term of the regression hyperplane, respectively, $\phi \left(z_{i}\right)$ is the nonlinear mapping function, C is the penalty coefficient, ε is the width of the insensitive tube, and $\xi_{i}$ and $\xi_{i}^{*}$ are slack variables. $z_{i}$ is the low-dimensional representation of the i-th sample, and $z_{j}$ is the low-dimensional representation of the j-th sample. Considering the high dimensionality, strong correlation, and nonlinearity of the spectral data, this study adopts the radial basis function (RBF) kernel function to construct the SVR model:

(9) $$K\left(z_{i},z_{j}\right)=exp{\left(-\gamma \left\|z_{i}-z_{j}\right\|\right)}^{2}$$

where γ controls the decay rate of the similarity between samples. Finally, the regression prediction function of SVR can be expressed as [25]:

(10) $$\hat{y}\left(z\right)=\sum_{i=1}^{n}\left(\alpha_{i}-\alpha_{i}^{*}\right)K\left(z_{i},z\right)+b$$

where $\hat{y}\left(z\right)$ is the predicted salinity value, and $\alpha_{i}$ and $\alpha_{i}^{*}$ are Lagrange multipliers.

## 3. Results

**Figure 5:**
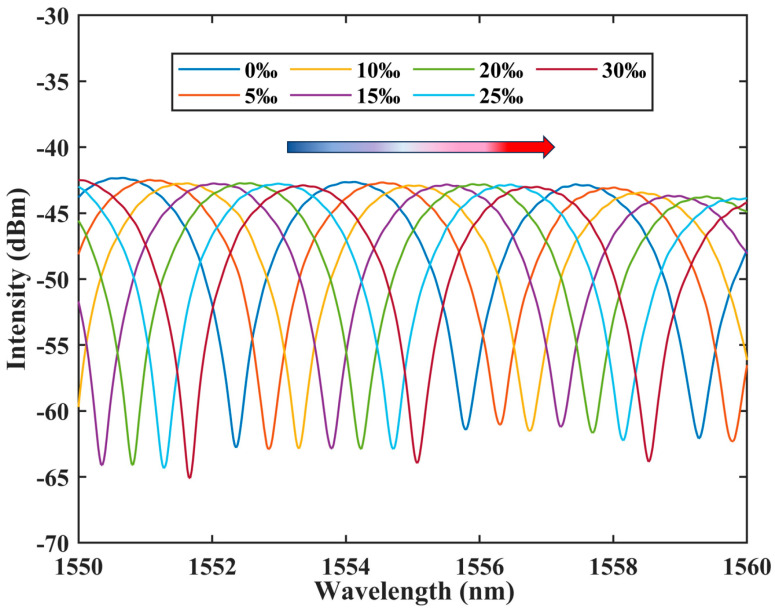
Spectral shift of the sensing system with salinity concentration under a BBS.

Figure 5 shows the spectral shift of the sensing system with salinity concentration under BBS obtained in the preliminary experiment. As illustrated, with salinity concentration gradually increasing from 0‰ to 30‰, the transmission spectrum of the SL exhibits a red shift toward longer wavelengths. This phenomenon originates from the increase in the solution refractive index caused by the rising salinity concentration, which in turn modulates the birefringence of the TPMF through the evanescent field, shifting the interference peaks toward longer wavelengths. The spectral drift trend is clear and continuous, with no obvious nonlinearity or jump, indicating that the sensor has good response consistency under broadband source excitation.

**Figure 6:**
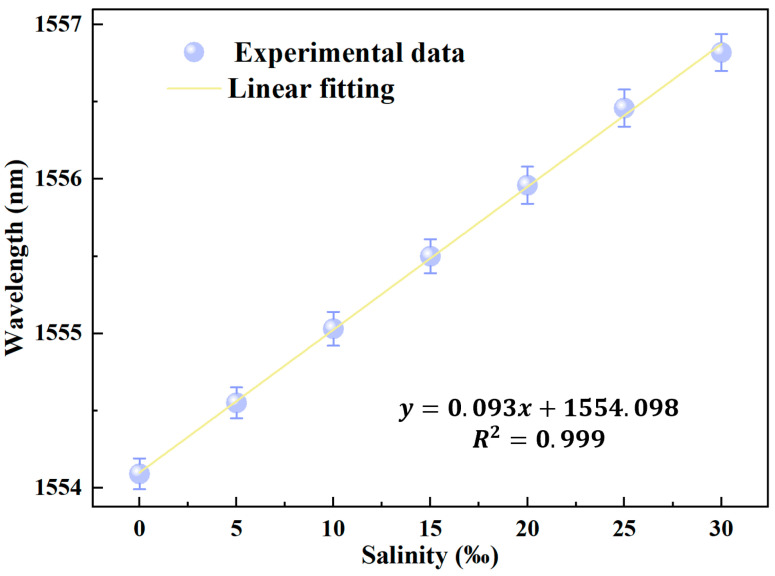
Linear fitting of the sensing system with salinity concentration under a BBS.

Figure 6 presents the linear fitting curve between salinity and the characteristic wavelength. Over the salinity range of 0‰ to 30‰, the characteristic wavelength increases linearly with salinity, and the fitted sensitivity is 93 pm/‰. The coefficient of determination (R^2^) reaches 0.999, demonstrating an excellent linear relationship between the wavelength shift and salinity. This linearity verifies the basic feasibility of salinity sensing under the BBS scheme. However, there remains room for further improvement in SNR and spectral bandwidth performance.

**Figure 7:**
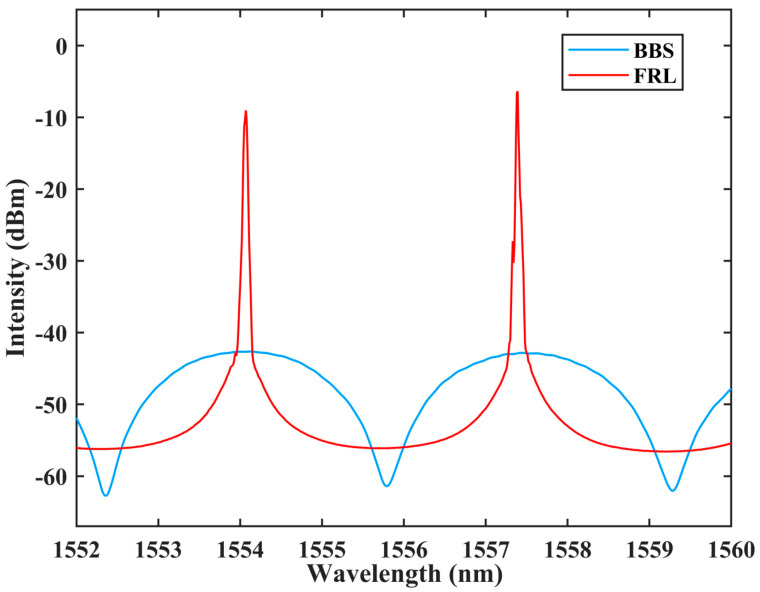
Comparison of output spectra between the BBS system and the FRL system at a salinity concentration of 0‰.

Figure 7 compares the spectra obtained when the SL is placed in a BBS system and in an FRL system at a salinity concentration of 0‰. As shown, the laser output spectrum of the FRL system exhibits two oscillation peaks. The positions of these two peaks precisely correspond to the peak wavelengths of two adjacent interference peaks in the SL transmission spectrum under the BBS, indicating that the laser wavelength of the FRL is locked to the interference peaks of the SL. More importantly, compared with the broad interference peaks under the BBS, the output spectral quality of the FRL system is significantly improved. The SNR increases from 22 dB to 54 dB, and the 3-dB bandwidth narrows from 1.75 nm to 0.06 nm. This characteristic of a narrow linewidth and high SNR lays a favorable spectral foundation for subsequent improvement of salinity measurement accuracy.

**Figure 8:**
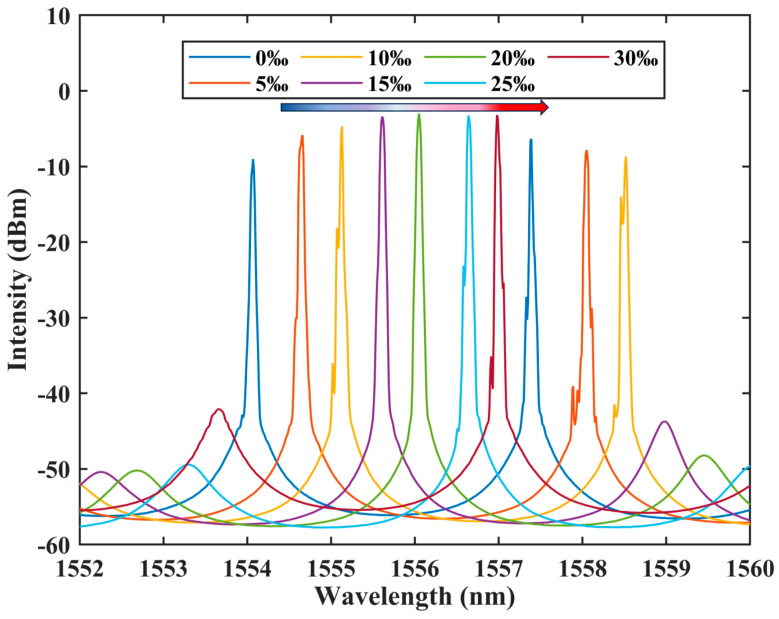
Spectral shift of the sensing system with salinity concentration under an FRL.

**Figure 9:**
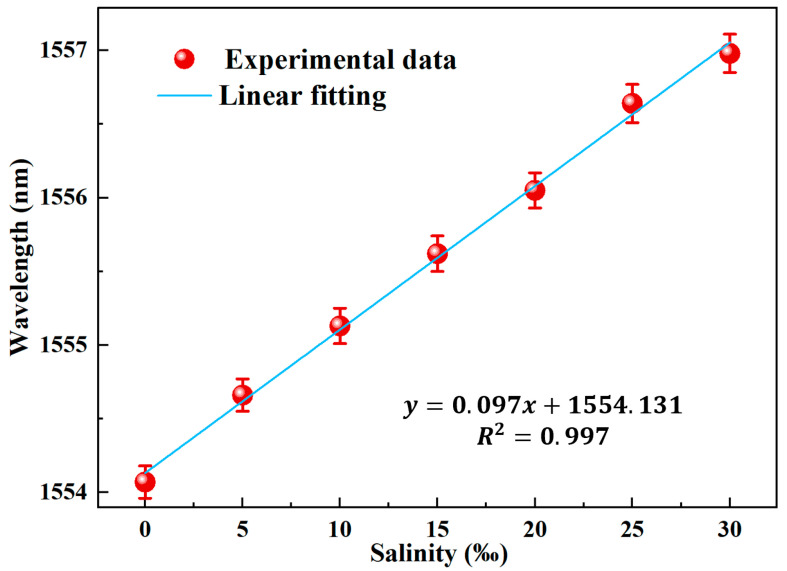
Linear fitting of the sensing system with salinity concentration under an FRL.

Figure 8 records the evolution of the output spectra of the FRL system at different salinity concentrations in this experiment. As the salinity gradually increases from 0‰ to 30‰, the wavelength position of the laser peak continuously shifts toward longer wavelengths, exhibiting a clear red-shift characteristic. In the low salinity range from 0‰ to 10‰, two laser peaks are observed in the output spectrum. Both peaks shift linearly with increasing salinity, maintaining the same drift rate. As salinity further increases, the dual-peak oscillation gradually transitions into single-peak operation due to the mode competition mechanism discussed earlier. Importantly, the linearity of the wavelength shift with salinity remains excellent throughout the entire salinity range, regardless of the number of oscillation peaks. The laser peak wavelengths at different salinity levels were extracted and subjected to linear regression analysis, and the results are shown in Figure 9. Over the measurement range of 0‰ to 30‰, the response of the wavelength to increasing salinity is highly linear, and the obtained sensitivity is 97 pm/‰. The coefficient of determination R^2^ reaches 0.993. The calculated MAE is 0.50‰, and the RMSE is 0.54‰. In Figure 6 and Figure 9, error bars indicate the standard deviation of five independent wavelength measurements at each salinity concentration. Moreover, owing to the inherent advantages of the narrow linewidth and high SNR of the laser spectrum, the random error in wavelength determination is smaller, thus providing a higher-quality data foundation for further improving the salinity prediction accuracy using the SVR algorithm.

**Figure 10:**
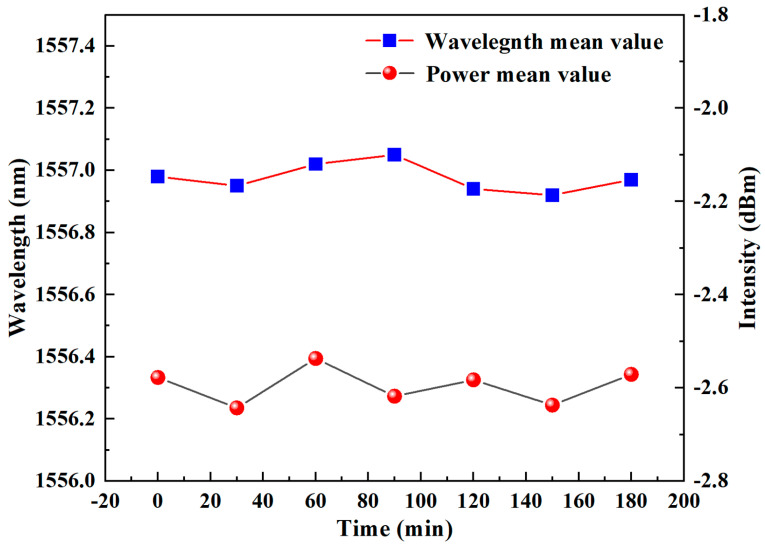
Wavelength stability and intensity stability of the output laser at a salinity concentration of 30‰.

Figure 10 presents the wavelength stability and intensity stability of the output laser at a salinity of 30‰. Within a three-hour test, the wavelength drift is less than 0.14 nm, and the intensity drift is less than 0.1 dB, which confirms the good stability of the proposed sensing system.

**Figure 11:**
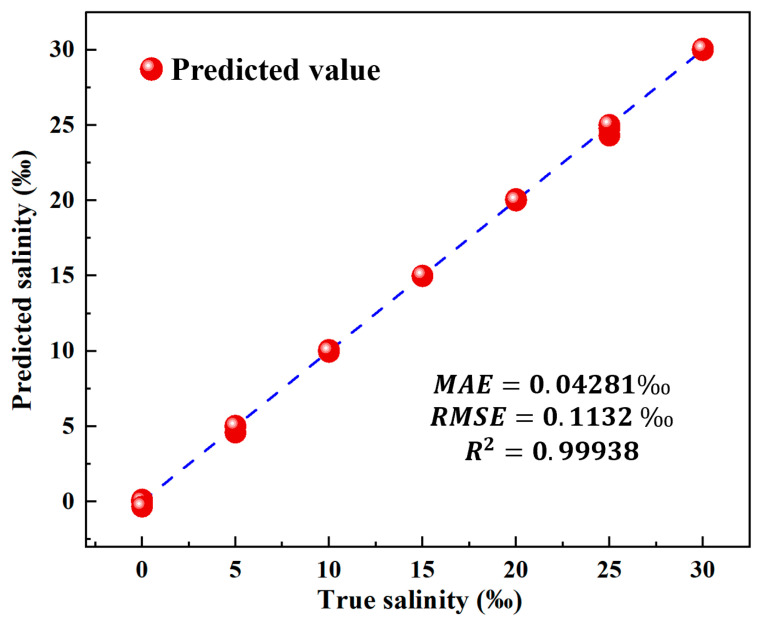
Comparison of actual and predicted values based on SVR algorithm.

Figure 11 presents a comparison between the predicted values from the SVR model and the actual values. As can be seen in the figure, the predicted values agree well with the actual values at almost all concentration levels. Quantitative evaluation metrics show that the R^2^ reaches 0.99938, the MAE is 0.04‰, and the RMSE is 0.11‰, indicating that the SVR model has extremely high prediction accuracy.

To ensure the reliability of the model evaluation, the dataset was randomly split into training, validation, and test sets with a fixed random seed for reproducibility. Moreover, the influence of data partitioning on prediction accuracy was examined by performing 10 independent runs with different random seeds. The resulting MAE and RMSE were 0.0431 ± 0.0012‰ and 0.1135 ± 0.0008‰, respectively, demonstrating that the SVR model is robust to random data splitting.

**Figure 12:**
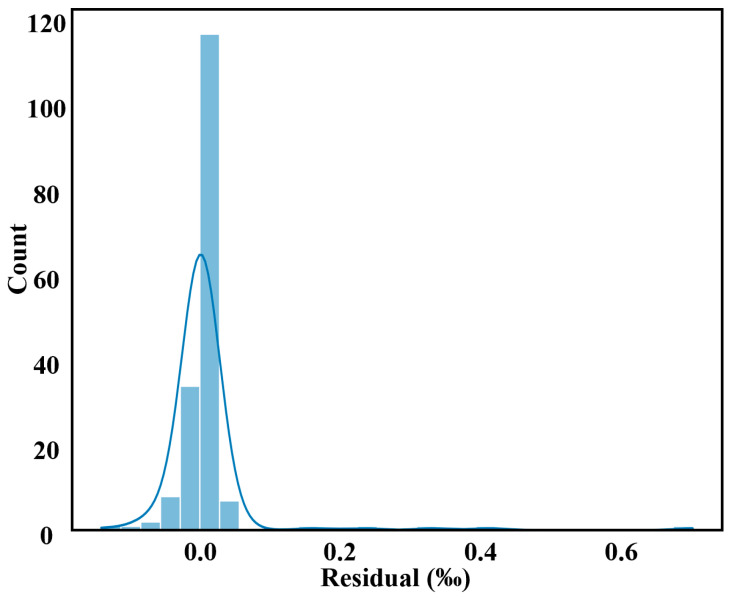
Residual distribution based on SVR algorithm.

Figure 12 shows the distribution of residuals between the predicted values and the true values after modeling with the SVR algorithm. It can be clearly observed in the figure that the residuals are mainly concentrated near zero, exhibiting a highly concentrated distribution pattern. Statistical results indicate that more than 99% of the sample prediction errors are within 0.01‰, with only a very small number of points exceeding this range. This distribution characteristic fully demonstrates that the SVR model possesses extremely high accuracy and a good generalization ability for salinity prediction.

**Figure 13:**
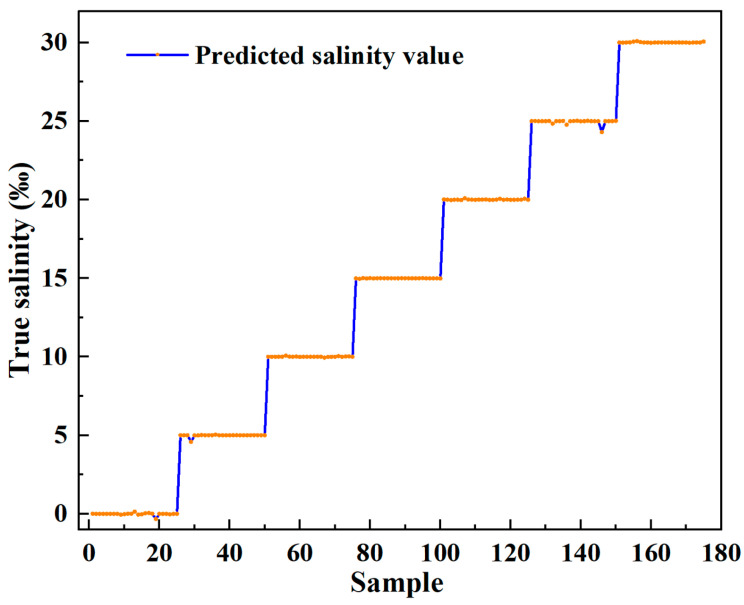
Predicted values of the test set samples.

Figure 13 presents the salinity values predicted by the SVR model for each sample. It can be observed that as the concentration gradient increases, the predicted values remain consistently stable without significant fluctuation. This result corroborates the phenomenon shown in Figure 11, where the residuals are tightly concentrated around zero. Table 1 compares the MAE and RMSE of four methods, namely conventional spectral demodulation, the Convolutional Neural Network (CNN) algorithm, the K-Nearest Neighbors (KNN) algorithm, and the proposed SVR. The results demonstrate that the SVR algorithm proposed in this work exhibits clear advantages in both MAE and RMSE over the other methods.

## 4. Discussion

It should be mentioned that mode competition occurs in the FRL due to the homogeneous gain broadening of the EDF [26], but it does not affect measurement accuracy. Within the dual peak range, both peaks shift linearly with salinity at the same rate, as shown in Figure 8. The laser wavelength follows the deterministic shift of the Sagnac interference peaks, so the change in peak number does not introduce nonlinearity or error. Notably, mode competition is often a drawback in conventional multi-wavelength EDFLs [27,28], but here it is harmless because the sensor relies on deterministic wavelength shifts, not on maintaining a fixed number of peaks. The dual-to-single-peak transition is spontaneous and requires no external intervention. This is further evidenced by the high linearity and the SVR-based accuracy, as presented in Figure 9 and Table 1.

In this experiment, it was observed that in the initial stage when the salinity increases from 0‰ to 10‰, the fiber ring laser outputs two stable laser peaks. As the salinity continues to increase, the two peaks gradually merge into a single peak. This is because as salinity increases, the entire transmission spectrum of the SL redshifts, causing the transmittance of one interference peak to decrease relatively, so its net gain falls below the lasing threshold. It should be emphasized that this evolution in the number of oscillation modes introduces only a negligible error in the measurement, which does not compromise the overall linearity or accuracy of the sensor. Experimental data show that within the dual-peak coexistence range, the drift rates of the two laser peaks with salinity are nearly identical. Therefore, even though the oscillation mode of the laser undergoes a natural transition, the linear mapping relationship between salinity and wavelength always holds, and the measurement accuracy of the sensor remains unaffected. This phenomenon purely originates from mode competition caused by the homogeneous broadening characteristics of the erbium-doped fiber, representing normal behavior of the laser under varying gain conditions. The transition from dual-peak to single-peak occurs spontaneously without the need for any external intervention.

Although the above mode transition does not impair the sensing performance, the presence of dual peaks may increase the complexity of signal processing in application scenarios that require single-laser output over the full measurement range. Future optimization can be achieved through passive design approaches, including shortening the length of the EDF to reduce the strength of mode competition, or appropriately increasing the pump power to force the lasing mode to converge toward the center of the gain spectrum. In addition, employing a shorter PMF with higher birefringence can also alter the transmission peak spacing of the SL such that the oscillation threshold is satisfied only for a single peak inside the cavity. These optimizations involve no movable or adjustable components and rely entirely on the passive selection of cavity parameters, thus introducing no additional instability.

The tapered waist diameter of 10.86 μm was selected through preliminary experiments [22,29]. This value provides a balance between high sensitivity and sufficient mechanical stability over the 0–30‰ salinity range. In addition, we should mention that temperature is known to have a significant influence on the SL spectrum, primarily causing a blue shift as temperature increases due to the thermo-optic effect and thermal expansion of the PMF. In this work, to exclude temperature-induced interference, all experiments were performed at a constant temperature of 25 °C. The main focus of this study was to demonstrate that the salinity discrimination capability was enhanced by the support vector regression algorithm, rather than to decouple temperature cross-sensitivity. Therefore, dedicated temperature monitoring was not performed, and the constant-temperature condition ensured that thermal effects were negligible. In future work, we will integrate a fiber Bragg grating into the sensor head to enable simultaneous measurement of salinity and temperature, and further combine machine learning algorithms to deepen the sensing technology.

The salinity measurement range in this work is limited to 0–30‰, whereas the practical salinity scale covers 2–42 PSU. This range was chosen because the main focus of this study is to validate the effectiveness of the SVR algorithm for improving accuracy in an FRL salinity sensor, rather than to cover the full natural salinity range. When salinity exceeds 30‰, the laser wavelength shifts further and the transmission peaks of the SL tend to overlap, making reliable single-peak tracking difficult with the current taper diameter and PMF length. Nevertheless, the 0–30‰ range is sufficient for many practical applications, such as estuarine and aquaculture monitoring, where salinity typically falls within this range. The present results successfully demonstrate that machine learning can significantly enhance sensing accuracy, and the same methodology can be applied to extended ranges in future work.

Another aspect for future improvement is the hardware-limited wavelength resolution. The current system uses an OSA with a maximum resolution of 0.01 nm. To overcome this limitation, a high-resolution interrogation method can be adopted. Specifically, a polarization analysis system consisting of a tunable laser, a polarization analyzer, and an optical power meter can be used to directly measure the transmission spectrum of the SL. In this approach, the tunable laser scans the wavelength range where the SL transmission peaks are located, the power meter records the transmission intensity at each wavelength step, and the polarization analyzer monitors the polarization state to eliminate polarization-induced errors. Such a system is capable of achieving a wavelength step size as small as 0.1 pm, thus providing a wavelength detection resolution of 0.1 pm. By interrogating the tapered-PMF-based SL with this setup, the transmission spectrum can be measured with a resolution of 0.1 pm instead of the OSA’s 10 pm resolution. This hardware upgrade would not require any change to the sensing head and could be combined with the SVR algorithm for even higher accuracy. Notably, state-of-the-art refractometers have demonstrated salinity resolutions down to 0.0001‱ [30] and 0.001‱ [31], indicating that our system’s hardware resolution is sufficient for coastal applications, but improvements in our system’s hardware resolution is required for deep sea applications.

This work adopts a continuous-wave FRL. It should be noted that mode-locked fiber lasers may offer additional advantages in certain sensing applications. Compared with continuous-wave lasers, a mode-locked laser can provide more longitudinal modes, which helps to further improve the SNR in certain demodulation systems [32,33]; meanwhile, a mode-locked laser can enable hybrid optical–radio-frequency demodulation, allowing multiple physical parameters to be mapped onto the repetition frequency or beat frequency of the laser [33]. Furthermore, it has been demonstrated that embedding an SL into a mode-locked laser cavity can overcome the traditional peak-tracking difficulties [34]. However, a mode-locked configuration requires the incorporation of a saturable absorber, which significantly increases system complexity and cost, and stable mode-locking is often difficult to maintain when the sensing head is exposed to the external environment. Given that the continuous-wave architecture adopted in this paper has already achieved a high measurement accuracy, exploration of a mode-locked scheme lies beyond the scope of this work, though it remains a promising direction for future research.

## 5. Conclusions

In this work, we have proposed and experimentally demonstrated a salinity sensing system based on an SL formed by a TPMF in an FRL. The system integrates the SL into an FRL cavity, which significantly improves the spectral quality by providing a narrow linewidth and SNR. Experimental results show that over the salinity range of 0‰ to 30‰, the sensor achieves a sensitivity of 97 pm/‰. Compared with the BBS, the ring laser cavity increases the SNR from 22 dB to approximately 54 dB and compresses the 3-dB bandwidth from 1.75 nm to 0.06 nm. By further adopting the SVR algorithm for linear regression modeling, the MAE is reduced from 0.50‰ to 0.04‰, representing a reduction factor of about 12, and the RMSE is reduced from 0.54‰ to 0.11‰, representing a reduction factor of about 4.7. R^2^ reaches 0.99988. To the best of our knowledge, this is the first work that combines salinity laser sensing with an artificial intelligence algorithm. The proposed system is expected to be valuable for aquaculture water quality management and estuarine ecological monitoring, where enhanced accuracy and good linearity are required.

## Figures and Tables

**Table 1:** Comparison of MAE and RMSE of different models in salinity prediction.

Demodulation Method	MAE (‰)	RMSE (‰)
Conventional spectral demodulation	0.5013	0.5362
CNN	0.0714	0.5976
KNN	0.0563	0.5307
SVR	0.0428	0.1132

## Data Availability

The data are not publicly available due to restrictions imposed by the funding institution and the need for further internal validation.
